# Baricitinib Inhibits the Aggressive Phenotype of Synovial Fibroblasts in Rheumatoid Arthritis via the JAK-STAT3-EGR1 Axis

**DOI:** 10.1007/s10753-026-02557-9

**Published:** 2026-07-08

**Authors:** Jing Wang, Yi Huang, Lingfei Mo, Fei Li, Xiaohao Wang, Yao Li, Ke Xu, Xinyi Liu, Hanchao Li, YuanYuan Li

**Affiliations:** 1 Department of Rheumatology and Immunology, First Affiliated Hospital of Xi’an JiaoTong University, Xi’an, 710061 China; 2 Biomedical Analysis Center, Army Medical University, Chongqing, China; 3 Xi’an Jiaotong University Affiliated HongHui Hospital, Xi’an, China

**Keywords:** Baricitinib, Rheumatoid arthritis, Synovial fibroblasts, JAK inhibition, EGR1

## Abstract

Synovial fibroblasts (SFs) transform into an aggressive phenotype in rheumatoid arthritis (RA), playing a significant role in sustaining synovitis and bone erosion. Baricitinib, a JAK inhibitor, has been validated as an effective treatment for RA. However, its direct inhibitory effects on RASFs remain poorly understood. The inflammatory response and aggressive behaviors (proliferation, apoptosis, migration, and invasion) of RASFs were assessed following treatment with varying concentrations of baricitinib. RNA sequencing was performed to identify differentially expressed genes and elucidate the impact of baricitinib on RASFs. The relationship between the JAK-STAT3 signaling pathway and downstream molecules (EGR1, BCL-2, MMP-2, MMP-9) was analyzed via western blotting. Collagen-induced arthritis (CIA) mice were treated with baricitinib via oral gavage or by intra-articular injection of shEGR1 to evaluate therapeutic efficacy. Baricitinib inhibited the production of inflammatory mediators in primary RASFs and reduced the progression of synovial inflammation and hyperplasia in RA patients as assessed by ultrasound. In vitro, baricitinib promoted apoptosis and inhibited the proliferation, migration, and invasion of RASFs, effects associated with decreased expression of the transcriptional regulator EGR1. In vivo, baricitinib effectively reduced arthritis scores, paw swelling, and the expression of inflammatory cytokines in the joint tissues of CIA mice. Similar therapeutic effects were observed upon inhibition of EGR1. Baricitinib inhibits the aggressive phenotype of RASFs through the JAK-STAT3-EGR1 axis, thereby contributing to the alleviation of the inflammatory response and joint destruction in RA.

## Introduction

Rheumatoid arthritis (RA) is a synovial disease involving immune dysregulation and a complex interaction between genetic and environmental factors [1]. The primary clinical manifestations include synovial inflammation, synovial hyperplasia and bone erosion [2]. Synovial fibroblasts are a key part of the hyperplastic synovial pannus and are overactivated in RA. Activated synovial fibroblasts contribute to aggressive behaviors such as invasion, angiogenesis, and matrix degradation of cartilage and bone by producing inflammatory cytokines, chemokines and matrix-degrading enzymes [3]. In light of the crucial role of SFs in the pathogenesis of RA, developing novel therapeutic strategies that target activated RASFs is of utmost importance [4, 5].

The Janus kinase (JAK) signal transducer and activator of transcription (STAT) pathway represents a key signaling hub in inflammatory and immune cells. Among the downstream STAT family members, STAT3 is particularly prominent in RASFs, where its phosphorylation leads to nuclear translocation and drives the expression of genes involved in cell survival (e.g., BCL-2), matrix remodeling (e.g., MMP-2, MMP-9), and inflammatory mediators [6]. Importantly, the immediate-early transcription factor EGR1 is a well-characterized STAT3 target gene that has recently emerged as a key regulator of synovial fibroblast aggression [7]. The inhibition of the JAK-STAT pathway with JAK inhibitors has been an important therapeutic strategy in chronic inflammatory diseases, such as RA and ankylosing spondylitis [8, 9]. Some JAK inhibitors have been approved for use as new therapies for RA, including tofacitinib, baricitinib and upadacitinib [10]. Baricitinib inhibits JAK1 and JAK2, thereby blocking cytokine signaling via the JAK-STAT pathway [11]. The efficacy and safety of baricitinib have been confirmed in clinical studies for RA patients with moderate-to-severe disease activity [12, 13]. These studies have shown that baricitinib not only reduces disease activity but also inhibits radiographic progression of structural joint destruction [14]. Current studies on the mechanism of baricitinib in treating RA mainly focus on achieving a new immune homeostasis through remodeling of the function and number of immune cells, thereby reducing the continuous progression of synovial inflammation [15–17]. However, evidence suggests that baricitinib can also act directly on RA synovial fibroblasts in vitro, inhibiting their aggressive behavior [18, 19]. Nevertheless, the impact of baricitinib on synovial cells has not been as extensively studied as its influence on immune homeostasis.

This study aimed to investigate the direct effects of baricitinib on RASFs and the underlying molecular mechanisms. We systematically examined its impact on the aggressive behaviors of RASFs, and elucidated the responsible signaling axis. To validate the translational relevance of our in vitro findings, we further employed a collagen-induced arthritis (CIA) mouse model. Our work seeks to provide a novel mechanistic insight into how baricitinib ameliorates synovial pathology, extending beyond its established immunomodulatory effects.

## Materials and Methods

### Patients and Specimens

Synovial tissue samples were obtained from five RA patients and five osteoarthritis (OA) patients (as controls) undergoing knee replacement surgery. These tissues were used for the primary isolation of synovial fibroblasts in subsequent in vitro experiments. Separately, a cohort of 23 RA patients, who had been treated with baricitinib for more than 3 months, were enrolled for clinical observation and ultrasound assessment. The baseline characteristics of the clinical cohort are summarized in Table 1. The study was conducted in accordance with the Declaration of Helsinki and was approved by the Medical Ethics Committee of the First Affiliated Hospital of Xi’an Jiaotong University (No. XJTUAF2023LSK-147). Written informed consent was obtained from all participants.

**Table 1:** Demographic and clinical characteristics of RA patients.

Characteristic	Before	After
Female, n(%)	16 (69.6%)	-
Age, years	43.5 ± 11.3	-
Disease duration, years	6.7 ± 3.2	-
RF positive, n(%)	22 (95.7%)	-
ACPA positive, n(%)	19 (82.6%)	-
RF and ACPA positive, n(%)	19 (82.6%)	-
CRP, mg/L	16.4 ± 9.5	1.7 ± 1.4
ESR, mm/h	36.1 ± 15.7	12.7 ± 6.7
No. tender joints	9.3 ± 6.3	1.2 ± 1.1
No. swollen joints	7.3 ± 4.8	1.0 ± 0.5
VAS scores	4.7 ± 2.1	1.5 ± 0.7
DAS28-CRP	5.2 ± 1.4	1.49 ± 0.73
DAS28-ESR	5.5 ± 1.6	1.86 ± 0.53
Patients using DMARDs		
Hydroxychloroquine, n(%)	19 (82.6%)	19 (82.6%)
Methotrexate, n(%)	17 (73.9%)	17 (73.9%)
Leflunomide, n(%)	11 (47.8%)	11 (47.8%)

### Synovial Fibroblasts Isolation, Culture and Treatment

Synovial tissue samples were washed with ice-cold PBS, minced, digested enzymatically and then seeded into tissue culture flasks for 24 h until the fragments attached to the plates in a mixture of DMEM supplemented with 10% fetal bovine serum in a CO_2_ incubator at 37 °C. Then, nonadherent cells were removed and adherent cells were cultured. After expansion to passages 3–5, the expression of CD90 on the cell surface was detected by flow cytometry, and cells with purity greater than 95% were used for subsequent functional assays and western blotting analyses. Once the cells had reached more than 80% confluency, they were treated with 1, 5 and 10 µM baricitinib (MedChemExpress) dissolved in DMSO (final DMSO concentration < 0.1%) for 12 h (for western blotting) or 24–72 h (for functional assays), or transfected with si-EGR1 sequences (target sequence: 5′-GAGCAUACCUGUAUUUGCU-3′; Genechem Co. Ltd.) using Lipofectamine 3000 at a final siRNA concentration of 50 nM into the cells for subsequent experiments.

### Ultrasound Assessment

Ultrasound-based joint assessment was performed in one or two of the most painful joints at baseline, with subsequent reassessment of the same joints after 12 weeks of baricitinib treatment. All ultrasound examinations were conducted by the same rheumatologist, who is certified in musculoskeletal ultrasound diagnosis, with standardized joint positions using the GE Logiq S8 ultrasound system (GE Healthcare, USA). The ultrasound scoring of affected joint synovitis for gray-scale and power Doppler was performed in accordance with the definition provided by Szkudlarek et al. [20].

### Quantitative Real-time PCR

Total RNA was isolated from RASFs using Trizol reagent (Invitrogen, USA), followed by cDNA synthesis with the TAKARA reverse transcription kit (Takara Biotechnology, China) as per the manufacturer’s instructions. The gene expression levels were evaluated by quantitative real-time polymerase chain reaction (PCR) using the ABI-7500 Thermal Cycler (Applied Biosystems, Foster City, CA, United States), and calculated using the 2^−ΔΔCt^ method. The primer sequences used for qRT-PCR were as follows: EGR1 5′-GGTCAGTGGCCTAGTGAGC-3′ and 5′-GTGCCGCTGAGTAAATGGGA-3′, MMP-2 5′-TACAGGATCATTGGCTACACACC-3′and 5′-GGTCACATCGCTCCAGACT-3′, MMP-9 5′- TGTACCGCTATGGTTACACTCG-3′and 5′-GGCAGGGACAGTTGCTTCT-3′, BCL-2 5′- GGTGGGGTCATGTGTGTGG-3′and 5′-CGGTTCAGGTACTCAGTCATCC-3′, GAPDH 5′- GGAGCGAGATCCCTCCAAAAT-3′and 5′-GGCTGTTGTCATACTTCTCATGG-3′.

### Western Blotting

Total proteins of RASFs were extracted with RIPA lysis buffer (Beyotime, China), and quantified by BCA protein assay kit (Beyotime). The proteins were separated using 10% SDS-PAGE and transferred onto a polyvinylidene fluoride membrane. Following the blocking step, in which 5% skim milk was used, the primary antibody for p-JAK1^Tyr1022^ (1:1000), STAT3 (1:1000), p-STAT3^Tyr705^ (1:1000), MMP-2 (1:500), MMP-9 (1:500) and β-actin (1:1000) (all from Beyotime, China), and BCL-2 (1:500) (Santa Cruz Biotechnology, USA) was added and incubated overnight at 4 °C. Following a wash with TBST, the membrane was incubated at room temperature with a solution of HRP-conjugated secondary antibody for one hour. Protein expression levels were quantified with an enhanced chemiluminescence detection kit (Beyotime) and normalized to β-actin, followed by analysis of the band integrated absorbance using ImageJ software.

### Cell Viability Assay

Following seeding in a 96-well plate at a density of 1 × 10⁴ cells per well, RASFs were treated with baricitinib or transfected with si-EGR1. At the indicated time points (24, 48, and 72 h), 10 µL of CCK-8 working solution (Beyotime) was administered to each well and the cells were incubated for 1 h under standard conditions (37 °C, 5% CO₂). Subsequently, the optical density at 450 nm was recorded with a BioTek Synergy LX microplate reader.

### Cell Apoptosis Detection

To evaluate apoptosis, RASFs were exposed to baricitinib or si-EGR1 for 48 h, harvested, and then stained with 5 µL of Annexin V-FITC and 5 µL of PI in 500 µL binding buffer according to the BD Biosciences manual for 15 min at room temperature in the dark. The stained cells were analyzed on a BD LSRFortessa TM flow cytometer, and the percentage of apoptotic cells was quantified using FlowJo V10.

### Wound Healing Assay

The RASFs were seeded in 6-well plates. Once the cells had reached a density of over 90%, they were scratched using pipette tips. The detached cells were then washed gently with PBS. Following the administration of baricitinib or si-EGR1 for 24 h, the wounded areas were quantified by measuring the distance of migration.

### Cell Invasion Assay

Prior to the invasion assay, 24-well Transwell inserts (Corning) with 8 μm pores were pre-coated with Matrigel (BD Biosciences). After pretreatment with baricitinib or si-EGR1, RASFs were harvested and resuspended in serum-free medium. A total of 4 × 10⁴ cells in 200 µL were seeded into the upper chamber, with the lower chamber containing 600 µL of 10% FBS-DMEM as a chemoattractant. After 12 h, cells that had invaded through the Matrigel were fixed, stained with 0.1% crystal violet, and counted in five random fields to determine the average invasion count.

### Collagen-induced Arthritis (CIA) Model and Therapeutic Interventions

CIA model induction: Arthritis was induced in 8-week-old male DBA/1J mice. On day 0, mice were immunized by an intradermal injection at the base of the tail with an emulsion containing 100 µg of bovine type II collagen (Chondrex, USA) and an equal volume of Freund’s complete adjuvant (Chondrex). A booster immunization was administered on day 21 near the primary site using the same amount of collagen emulsified in Freund’s incomplete adjuvant.

Experimental groups and treatment: Mice with established arthritis (clinical onset typically after day 25) were randomly assigned to five groups (*n* = 7 per group): (1) CIA + Vehicle: CIA model mice receiving daily oral gavage of PBS; (2) CIA + Baricitinib: CIA mice receiving daily oral gavage of baricitinib (20 mg/kg, suspended in PBS) from day 21 until endpoint; (3) CIA + AAV-scramble Control: CIA mice receiving a single intra-articular injection of control AAV (AAV-scramble, 1 × 10¹² viral genomes in 10 µL) into the knee joint on day 7, plus daily intraperitoneal injection of PBS; (4) CIA + AAV-shEGR1: CIA mice receiving a single intra-articular injection of AAV-shEGR1 (1 × 10¹² viral genomes in 10 µL) into the knee joint under isoflurane anesthesia using a 30-gauge needle on day 7; (5) CIA + ML264: CIA mice receiving daily intraperitoneal injection of the EGR1 inhibitor ML264 (10 mg/kg). One independent control group was set up: Naïve Control: Age-matched, non-immunized healthy mice receiving daily oral gavage of PBS.

Clinical and pathological evaluation: Arthritis severity was assessed every 2–3 days by two independent observers blinded to the treatment groups. A clinical score (0–4 per paw) was used: 0, normal; 1, mild erythema/swelling in tarsals or ankle; 2, mild erythema/swelling from ankle to tarsals; 3, moderate swelling from ankle to metatarsals; 4, severe widespread swelling/erythema or ankylosis. The sum of scores from all four paws constituted the total arthritis score. Paw thickness was measured with a digital caliper. On day 49, mice were euthanized. Knee and ankle joints were harvested for µCT imaging, histological processing, and RNA extraction. For histology, joints were fixed in 4% paraformaldehyde, decalcified in 0.5 M EDTA (pH 7.4) for 2–3 weeks, and embedded in paraffin.

### µCT and Histological Analysis

Mouse knee joints were collected and dissected to remove soft tissues, followed by fixation of the remaining structures in 10% formaldehyde. High-resolution micro-computed tomography (µCT) was performed using a Skyscan 1172 system for imaging. Image analysis and three-dimensional reconstruction were carried out with CTAn v1.9 and NRecon v1.6 software. For histological evaluation, the fixed joints underwent decalcification in Osteodec (Bioptical, Italy) for 24 h, then were dehydrated and embedded in paraffin. Tissue sections were stained with hematoxylin and eosin (H&E) for morphological examination. Immunohistochemical staining for EGR1 expression in the synovial membrane was performed on paraffin-embedded sections using an anti-EGR1 antibody (Santa Cruz Biotechnology, USA).

### Statistical Analysis

Data were statistically analyzed using SPSS 23.0 (SPSS, Inc, Chicago, IL). Continuous data are expressed as mean ± standard deviation, and categorical data as frequency (percentage). For comparisons involving more than two groups, one-way ANOVA with Dunnett’s post hoc test was applied. Differences between two groups were evaluated using the t-test. Where data deviated from normality, nonparametric tests were used. A two-tailed P-value below 0.05 was considered statistically significant.

## Results

### Baricitinib Suppressed the Inflammatory Response of RASFs

**Fig. 1:**
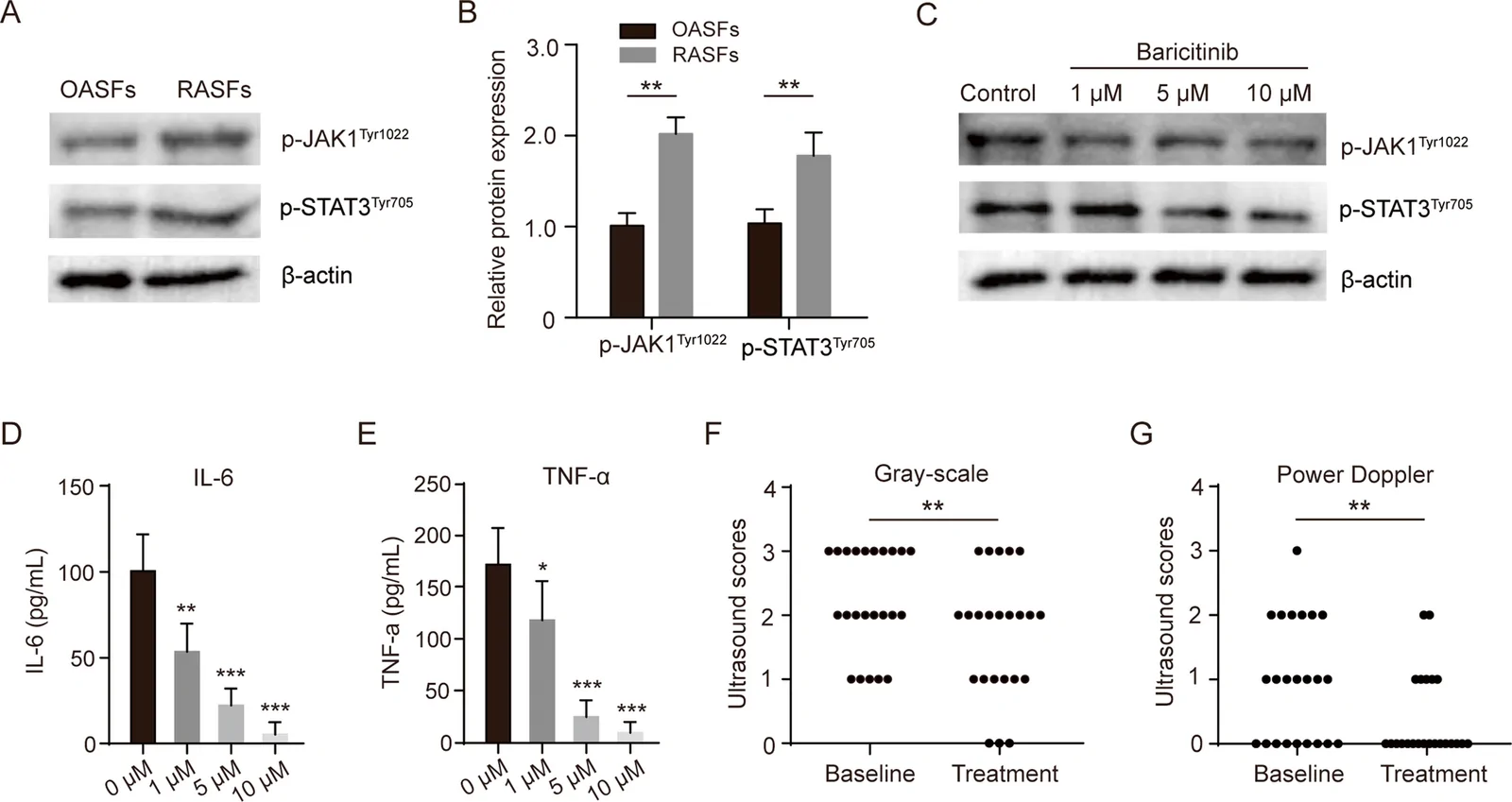
Baricitinib suppressed the inflammatory response of RASFs. (**A**-**B**) Protein levels of p-JAK^Tyr1022^ and p-STAT3^Tyr705^ in OASFs (*n* = 5) or RASFs (*n* = 5) were analyzed by western blotting. (**C**) Levels of p-JAK1^Tyr1022^ and p-STAT3^Tyr705^ in RASFs treated with baricitinib. (**D**-**E**) Secretion of IL-6 and TNF-α into the culture medium of RASFs treated with baricitinib, as measured by ELISA. (**F**-**G**) Ultrasound scores (grey-scale and power Doppler) for synovitis in the most affected joints of RA patients (*n* = 23) at baseline and after 3 months of baricitinib treatment. Data are expressed as mean ± SD. ^*^P < 0.05, ^**^P < 0.01, ^***^P < 0.001.

To determine whether the JAK-STAT3 pathway is activated in synovial fibroblasts from patients with RA, we isolated synovial fibroblasts from the knees of RA patients and OA patients. Compared with osteoarthritis SFs (OASFs), the phosphorylation levels of JAK1 and STAT3 were significantly increased in RASFs, suggesting an important role of the JAK-STAT3 pathway in RA pathogenesis (Fig. 1A-B). We next treated RASFs with different concentrations of baricitinib in vitro for 12 h and found that the expression levels of p-JAK1 and p-STAT3 were significantly decreased in a dose-dependent manner (Fig. 1C). In addition, the secretion of IL-6 and TNF-α in the culture medium was also significantly reduced by ELISA detection (Fig. 1D-E). Consistent with the in vitro findings, ultrasound scores for both grey-scale and power Doppler synovitis markedly decreased in the most affected joints of RA patients following 3 months of baricitinib treatment (Fig. 1F, G).

### Baricitinib Inhibited the Aggressive Behavior of RASFs

**Fig. 2:**
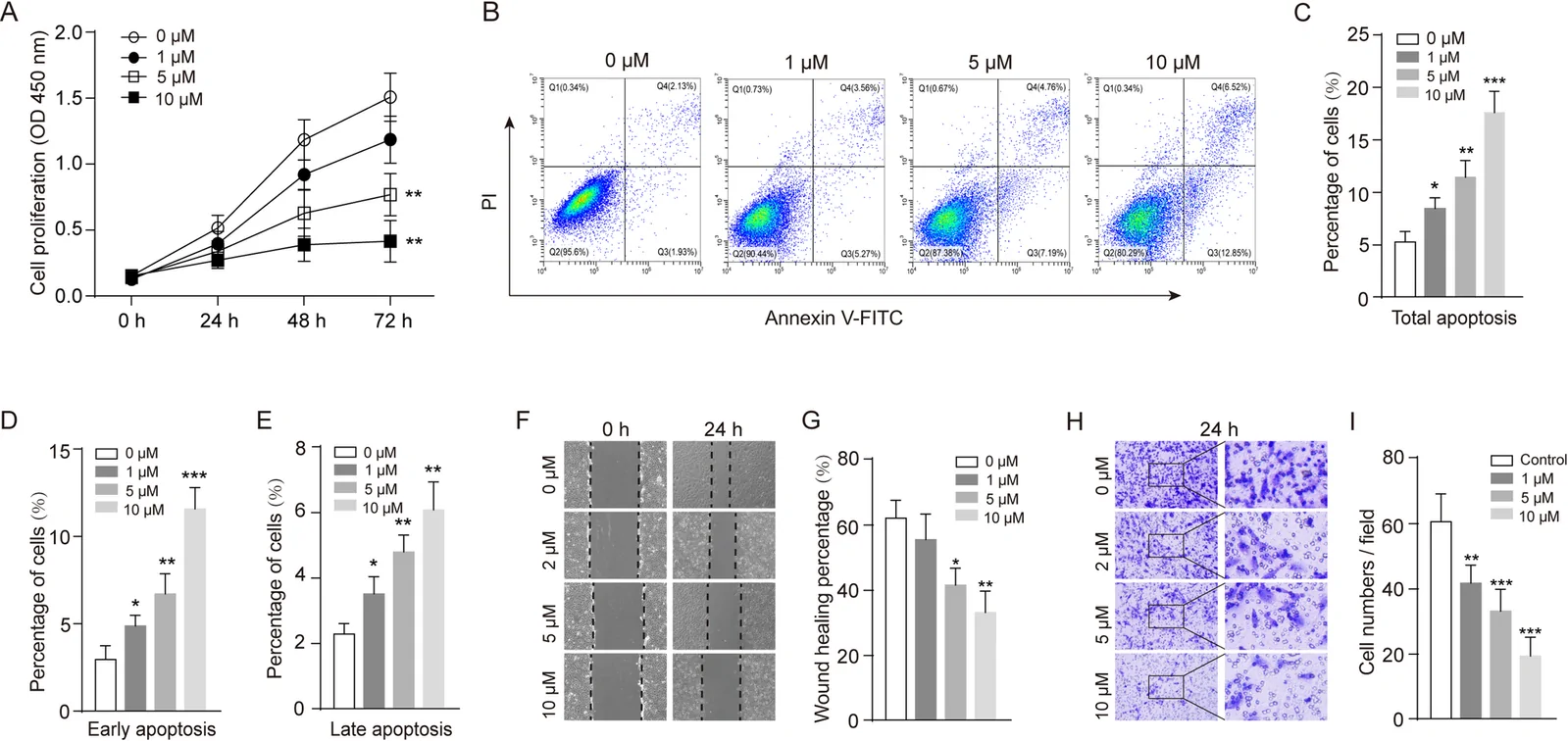
Baricitinib promoted apoptosis and inhibited proliferation, migration and invasion of RASFs. (**A**) Cell viability of RASFs treated with baricitinib was assessed by CCK-8 assay. (**B**–**E**) Apoptosis of RASFs treated with baricitinib was analyzed by Annexin V-FITC/PI staining and flow cytometry. (**F**-**G**) Migration of RASFs treated with baricitinib was evaluated by wound healing assay. The wound closure rate is presented. (**H**-**I**) Invasion of RASFs treated with baricitinib was evaluated by Transwell assay. Data are expressed as mean ± SD. ^*^P < 0.05, ^**^P < 0.01, ^***^P < 0.001.

To investigate the antiproliferative activity of baricitinib, RASFs were treated with baricitinib for 24, 48, and 72 h, and cell viability was measured by the CCK-8 assay. Baricitinib inhibited the growth of RASFs in a time-dependent manner (Fig. 2A). We further explored whether baricitinib could inhibit cell proliferation by inducing apoptosis in RASFs. Flow cytometry revealed that the proportion of apoptotic cells were increased in the baricitinib-treated RASFs (Fig. 2B-C), with a dose-dependent rise in both early and late apoptotic populations (Fig. 2D-E). Furthermore, baricitinib remarkably suppressed the migration (Fig. 2F-G) and significantly inhibited the invasion (Fig. 2H-I) of RASFs compared to the control. Taken together, these data indicate that baricitinib inhibits the aggressive behavior characteristic of RASFs in vitro.

**Fig. 3:**
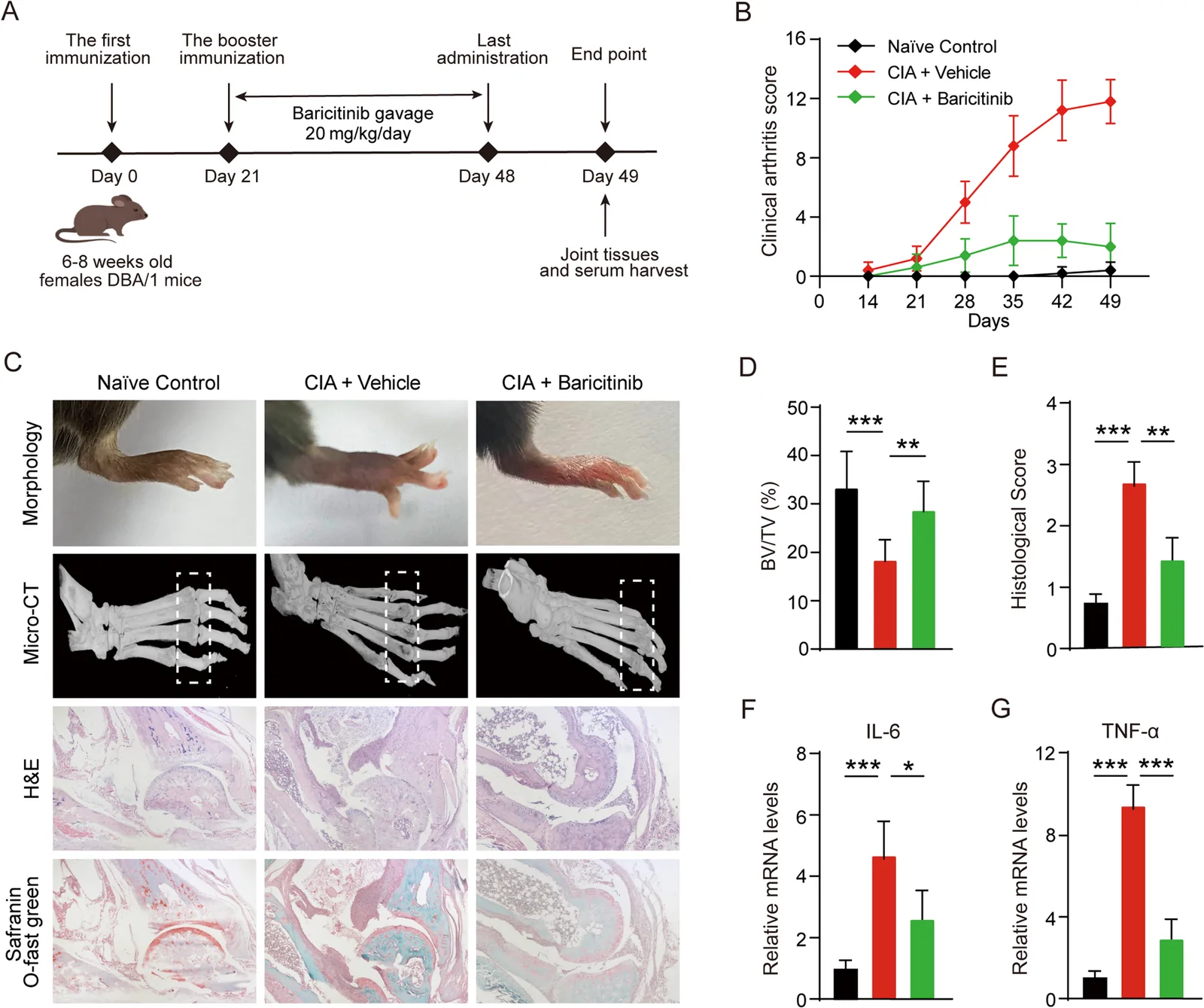
Baricitinib prevents arthritis progression and attenuates severity in CIA mice. (**A**) Experimental timeline for baricitinib treatment in the CIA model. (**B**) Clinical arthritis scores in naïve control mice, vehicle-treated CIA mice, and baricitinib-treated CIA mice (*n* = 7 per group). (**C**-**E**) The micro-CT images and histological evaluation of paws demonstrated the destruction of the bone and cartilage in CIA mice. (**F**-**G**) mRNA expression of *IL-6* and *TNF-α* in knee joint tissues analyzed by qRT-PCR. Data were expressed as mean ± SD. ^*^P < 0.05, ^**^P < 0.01, ^***^P < 0.001.

To evaluate the in vivo relevance of these findings, we employed a collagen-induced arthritis (CIA) model in DBA/1J mice. Baricitinib (20 mg/kg/day) was administered by oral gavage from day 21 (post-booster immunization) until day 48 (Fig. 3A). Compared with the naïve control mice without collagen injection, the arthritis score of CIA mice increased significantly over time, while baricitinib significantly decreased the joint score of mice at the same time (Fig. 3B). Micro-CT imaging and histopathological analysis demonstrated that baricitinib markedly prevented bone loss and erosion (Fig. 3C-E). Consistent with its anti-inflammatory effects in vitro, qRT-PCR analysis of knee tissues showed that baricitinib significantly suppressed the mRNA expression of IL-6 and TNF-α (Fig. 3F-G).

### Transcriptomic Profiling Reveals Baricitinib-induced Gene Expression Changes in RASFs

**Fig. 4:**
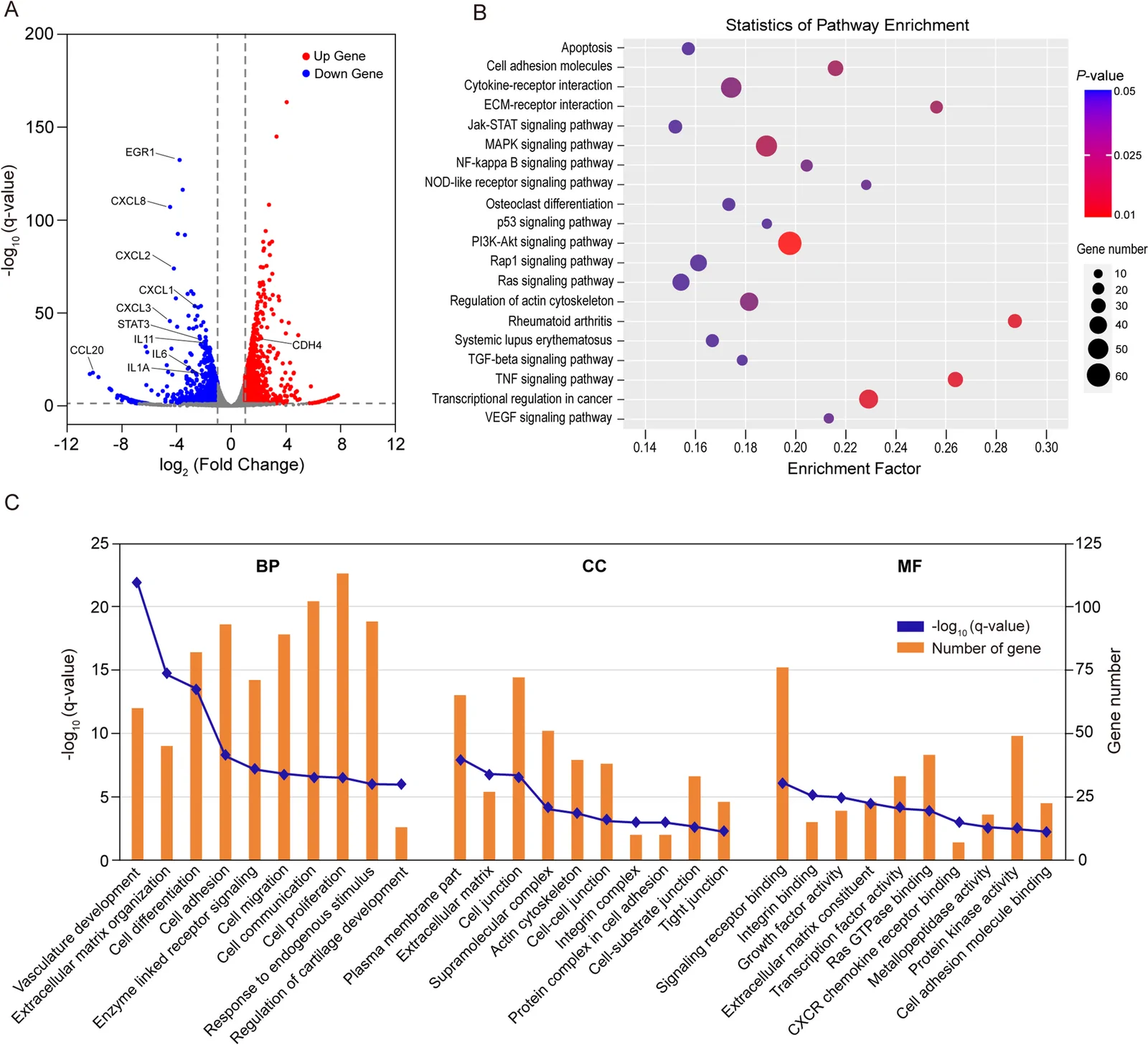
Transcriptomic profiling of baricitinib-treated RASFs. (**A**) Volcano plot of differentially expressed genes (DEGs). Downregulated and upregulated genes are shown in blue and red, respectively. (**B**) Significantly enriched KEGG pathways among the DEGs. The dot size represents the number of genes; the color indicates the statistical significance. (**C**) The top 10 enriched Gene Ontology (GO) terms in biological process (BP), cellular component (CC), and molecular function (MF) categories.

To elucidate the molecular mechanisms underlying baricitinib’s effects, we performed RNA sequencing (RNA-seq) on RASFs treated with 10 µM baricitinib. Comparative analysis identified 2,131 differentially expressed genes (DEGs), with 985 downregulated and 1,146 upregulated (Fig. 4A). Notably, many downregulated genes encode key inflammatory mediators and chemokines, such as IL1A, IL6, CXCL1, and CXCL2. Gene set enrichment analysis using DAVID highlighted significant KEGG pathways, including rheumatoid arthritis, TNF signaling, and ECM-receptor interaction (Fig. 4B). Pathways like JAK-STAT and PI3K-AKT signaling were also enriched, aligning with baricitinib’s known mechanism and its broader cellular impact. Gene Ontology (GO) analysis showed that the DEGs were significantly enriched in biological processes related to cell proliferation and adhesion, cellular components including the plasma membrane and supramolecular complexes, and molecular functions such as signaling receptor binding and transcription factor activity (Fig. 4C). Of particular interest was the marked downregulation of the immediate-early transcription factor EGR1, given its known role in regulating inflammation, cell survival, and matrix remodeling. This focused our subsequent mechanistic investigation on the JAK-STAT3-EGR1 axis.

### Baricitinib Regulates RASFs Function via the JAK-STAT3-EGR1 Axis

**Fig. 5:**
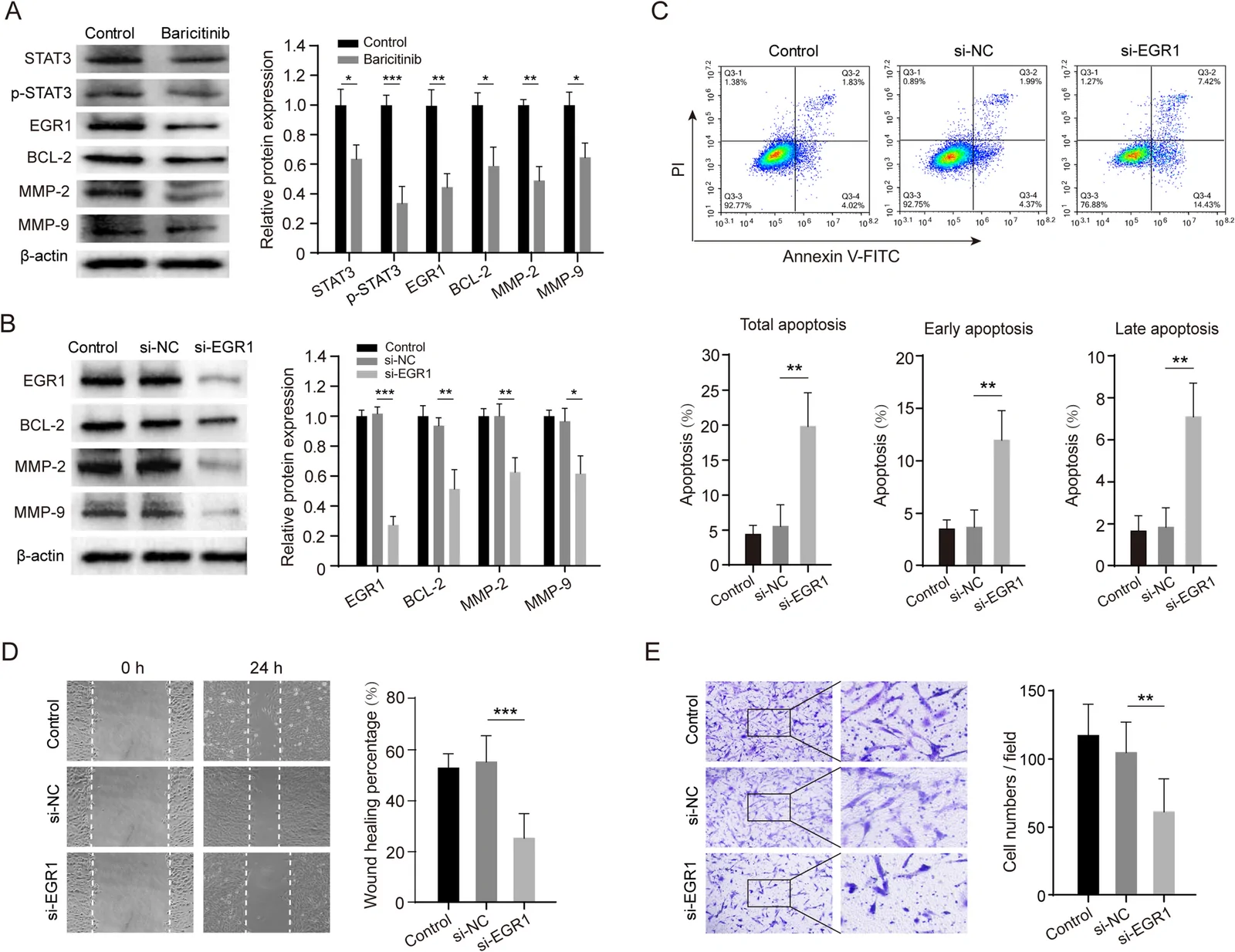
Baricitinib regulates synovial cell function by inhibiting the JAK-STAT3-EGR1 axis. (**A**) Protein levels of STAT3, p-STAT3^Tyr705^, EGR1, BCL-2, MMP-2 and MMP-9 in RASFs treated with 10 µM baricitinib. (**B**) Protein levels of EGR1, BCL-2, MMP-2 and MMP-9 in RASFs transfected with si-EGR1. (**C**) Apoptosis of RASFs transfected with si-EGR1 were analyzed by Annexin V-FITC/PI staining. (**D**) Migration of RASFs transfected with si-EGR1 was evaluated by wound healing assay. (**E**) Invasion of RASFs transfected with si-EGR1 was evaluated by Transwell assay. Data were expressed as mean ± SD. ^*^P < 0.05, ^**^P < 0.01, ^***^P < 0.001.

Given that EGR1 is a transcription factor often regulated by STAT3 and involved in cell survival and invasion, we hypothesized that the JAK-STAT3-EGR1 axis might mediate the effects of baricitinib. To test this, we first confirmed by western blotting that baricitinib treatment concurrently reduced the levels of phosphorylated STAT3 (p-STAT3), EGR1, and the downstream effector proteins BCL-2, MMP-2, and MMP-9 (Fig. 5A). This established a correlative link between pathway inhibition and the expression of key functional proteins. We next asked whether EGR1 downregulation alone was sufficient to mimic baricitinib’s effects. Transient transfection of si-EGR1 into RASFs significantly reduced the protein levels of BCL-2, MMP-2, and MMP-9 (Fig. 5B), paralleling the effects of baricitinib. Moreover, EGR1 knockdown markedly promoted apoptosis (Fig. 5C) and inhibited both migration and invasion (Fig. 5D-E) in RASFs. Collectively, these data indicate that EGR1 is a critical downstream mediator of the JAK-STAT3 pathway in RASFs. The finding that baricitinib inhibits this axis while EGR1 knockdown phenocopies key drug effects strongly supports a model where baricitinib suppresses RASFs aggression through inhibition of the JAK-STAT3-EGR1 signaling cascade.

### EGR1 Inhibition Alleviates Disease severity in CIA Mice

**Fig. 6:**
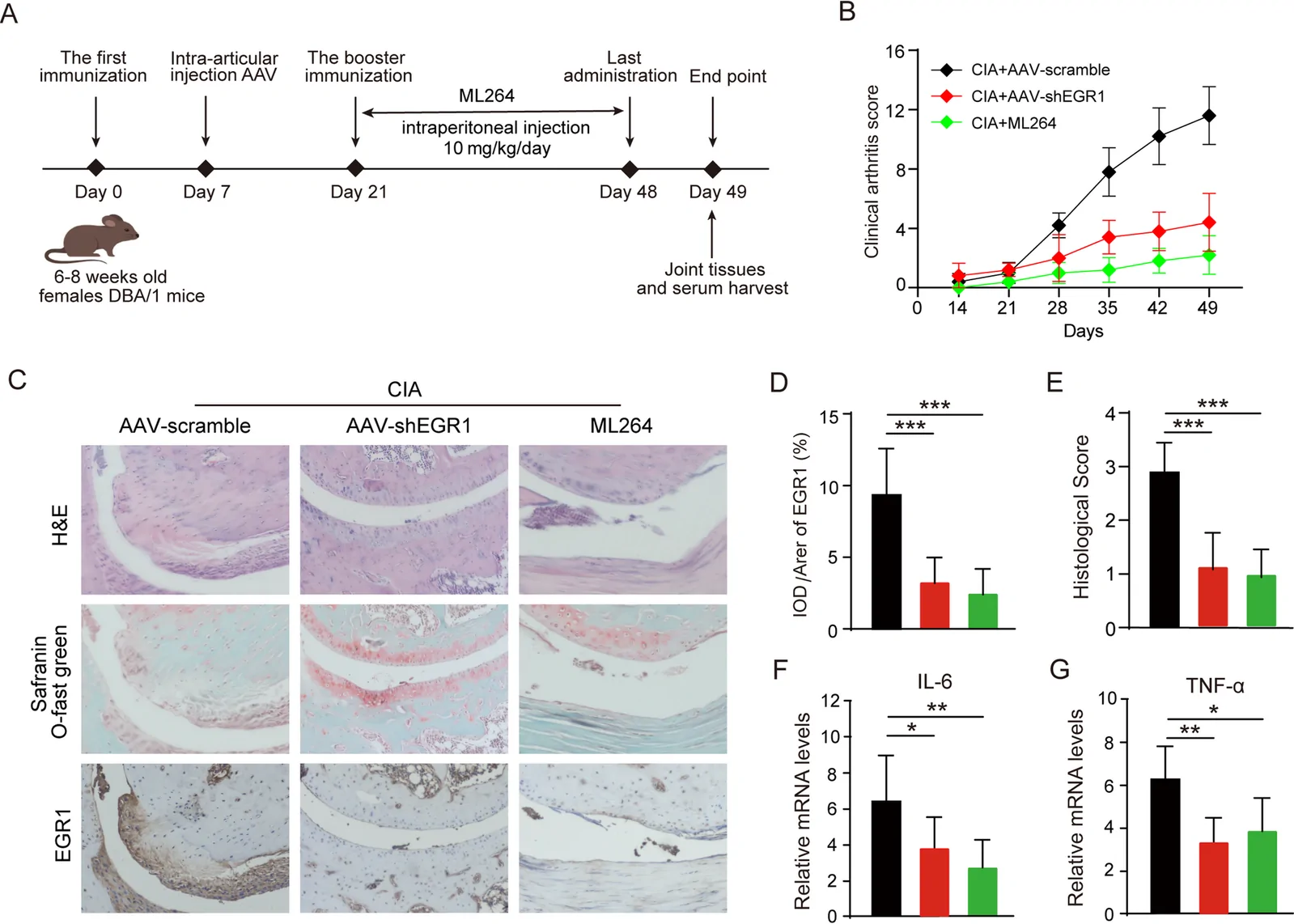
Inhibition of EGR1 expression alleviates arthritis in CIA mice. (**A**) Experimental timelines for AAV-shEGR1 intra-articular injection and ML264 intraperitoneal administration in the CIA model. (**B**) Clinical arthritis scores over time in CIA mice treated with AAV-shEGR1, ML264, or the corresponding controls. (**C**-**E**) Histological, immunohistochemical, and scoring analysis of joint pathology. (**F**-**G**) mRNA expression of *IL-6* and *TNF-α* in knee joint tissues. Data were expressed as mean ± SD. ^*^P < 0.05, ^**^P < 0.01, ^***^P < 0.001.

To assess the therapeutic potential of targeting EGR1 in vivo, we inhibited EGR1 in CIA mice using two complementary approaches: local knockdown via intra-articular injection of AAV-shEGR1 on day 7, or systemic pharmacological inhibition via daily intraperitoneal injection of ML264 (10 mg/kg) from day 21 to 48 (Fig. 6A). CIA mice in the vehicle control group developed progressive arthritis, which was significantly ameliorated by both AAV-shEGR1 and ML264 treatments (Fig. 6B). Immunohistochemistry confirmed effective reduction of EGR1 protein in the synovium by both interventions (Fig. 6C-D). Consistent with the improved clinical scores, histological analysis demonstrated that EGR1 inhibition markedly reduced inflammatory cell infiltration, cartilage degradation, and bone erosion (Fig. 6C-E). At the molecular level, qRT-PCR analysis of knee tissues showed that AAV-shEGR1 and ML264 significantly suppressed the mRNA expression of the pro-inflammatory cytokines IL-6 and TNF-α (Fig. 6F-G).

## Discussion

Joint destruction is a defining feature of RA. In rheumatoid joints, synovial fibroblasts (SFs) undergo exponential expansion, transforming the synovial lining into an invasive, hyperplastic tissue that drives joint damage [21]. The enhanced survival of these cells is underpinned by elevated expression of anti-apoptotic genes like BCL2 and MCL1 [22]. Given that the JAK-STAT3 pathway regulates these genes, we postulated that the JAK inhibitor baricitinib could induce apoptosis in RASFs. The JAK-STAT pathway is a primary mediator of cytokine signaling [23]. Thus, JAK inhibitors are thought to primarily modulate the reactive responses of RASFs to inflammatory cytokines. Whether they directly induce apoptosis remains debated. While some reports indicate JAK inhibitors alone do not trigger apoptosis [24], possibly requiring concomitant stress signals, others demonstrate direct pro-apoptotic effects in vitro [25]. Notably, RASFs exhibit constitutively active JAK-STAT signaling [26], a feature we confirmed. Our findings demonstrate that baricitinib directly inhibits this basal JAK-STAT3 activation in RASFs, reducing IL-6 and TNF-α secretion and synovitis progression in patients. This suggests that targeting intrinsic JAK-STAT activity is a viable strategy against RASFs.

In the context of RA, synovial fibroblasts activate the JAK-STAT signaling pathways in response to exposure to RA synovial fluid, which was found to contain a multitude of immune cell-derived cytokines, in addition to autocrinally produced interleukin-6 (IL-6), type I interferons, and other factors [22]. Under the stimulation of these inflammatory mediators, synovial fibroblasts secreted metalloproteinases (MMP-1, MMP-9 and others) and chemokines to degrade extracellular matrix components and promote invasion [27, 28]. The JAK-STAT pathway was identified as a crucial pathway for RASFs in inducing cartilage erosion through the secretion of MMPs. In our study, another significant finding was that baricitinib inhibited RASFs migration and invasion. In vitro studies, it has found that other JAKi reduced the secretion of MMP-2, MMP-9 and other metalloproteinases by RASFS [29], thus inhibiting cell migration and invasion, which was consistent with our results. Furthermore, we found that the levels of CXCL1, CXCL2, CXCL3, CXCL8 and other chemokines were also decreased in transcriptomic sequencing of RASFs after baricitinib treatment. These data may provide insight into the pharmacological mechanism of baricitinib in inhibiting RASFs invasion of cartilage and bone from another perspective.

Unlike previous studies of JAK inhibitors focusing on their inhibitory effects on the JAK-STAT pathway, recent studies have shown that JAK inhibitors can also inhibit the activated MAPK pathway in RASFS, such as the activation of ERK, p38, and JNK1/2 [30]. We further analyzed RASFs transcriptome data treated with baricitinib and found that in addition to the JAK-STAT pathway, baricitinib also affected several signaling pathways such as MAPK, NF-kB, NOD, P53, PI3K-AKT, TGF-b, TNF and VEGF. Suppression of the above involved cytokine-induced responses in RASFs may terminate the cytokine-driven vicious cycles between cytokines and RASFs [22]. In our RNA-Seq data, baricitinib significantly downregulated EGR1 expression in RASFs. EGR1 is a transcription factor that has been implicated in a variety of pathological processes, including tissue injury, immune response and fibrosis [31, 32]. Recent studies have shown that EGR1 is participated in tumor cell proliferation, invasion, and metastasis and in tumor angiogenesis, which are associated with cancer initiation and progression [33]. In RASFs, EGR1 was overexpressed compared with OA or healthy controls. Although EGR1 was involved in the activation of RASFs, the functional consequences of EGR1 overexpression were still unclear. It has been reported that EGR1 can induce the expression of E-cadherin transcription inhibitors to participate in tumor invasion and metastasis [34], and that EGR1 can trigger MMP1 expression by directly binding to its promoter region [35]. Since the transcription factor activity of EGR1 may control the levels of several tissue inhibitor of metalloproteinases genes, EGR1 may act as a molecular switch that is part of the complex process that regulates MMP activities in arthritis [36]. In vitro experiments, we confirmed that EGR1 inhibition reduced the expression of BCL-2 and MMP-2 in RASFs, promoted apoptosis of RA cells, and reduced cell migration. In addition, injections of AAV-shEGR1 into the joints or intraperitoneal injection of ML264 also effectively alleviated arthritis symptoms of CIA mice. Collectively, our findings demonstrate the pathogenic function of EGR1 in RA and highlight its potential as a novel therapeutic target.

Several limitations of this study should be acknowledged. First, while baricitinib inhibits both JAK1 and JAK2, the specific contribution of each isoform to EGR1 downregulation remains unclear. Future studies using highly selective JAK inhibitors will help delineate the respective roles of JAK1 versus JAK2 in modulating EGR1 and the aggressive RASF phenotype. Second, the baricitinib dose used in our CIA model is higher than the standard clinical regimens. Although this dose is within the established safety margin and is commonly used in murine arthritis models, the extent to which clinical dosing achieves sufficient synovial tissue drug concentrations to suppress the JAK-STAT3-EGR1 axis remains to be determined. Future pharmacokinetic studies in human synovial tissue or imaging-based assessments of target engagement would help bridge the gap between our in vivo findings and clinical translation.

**Fig. 7:**
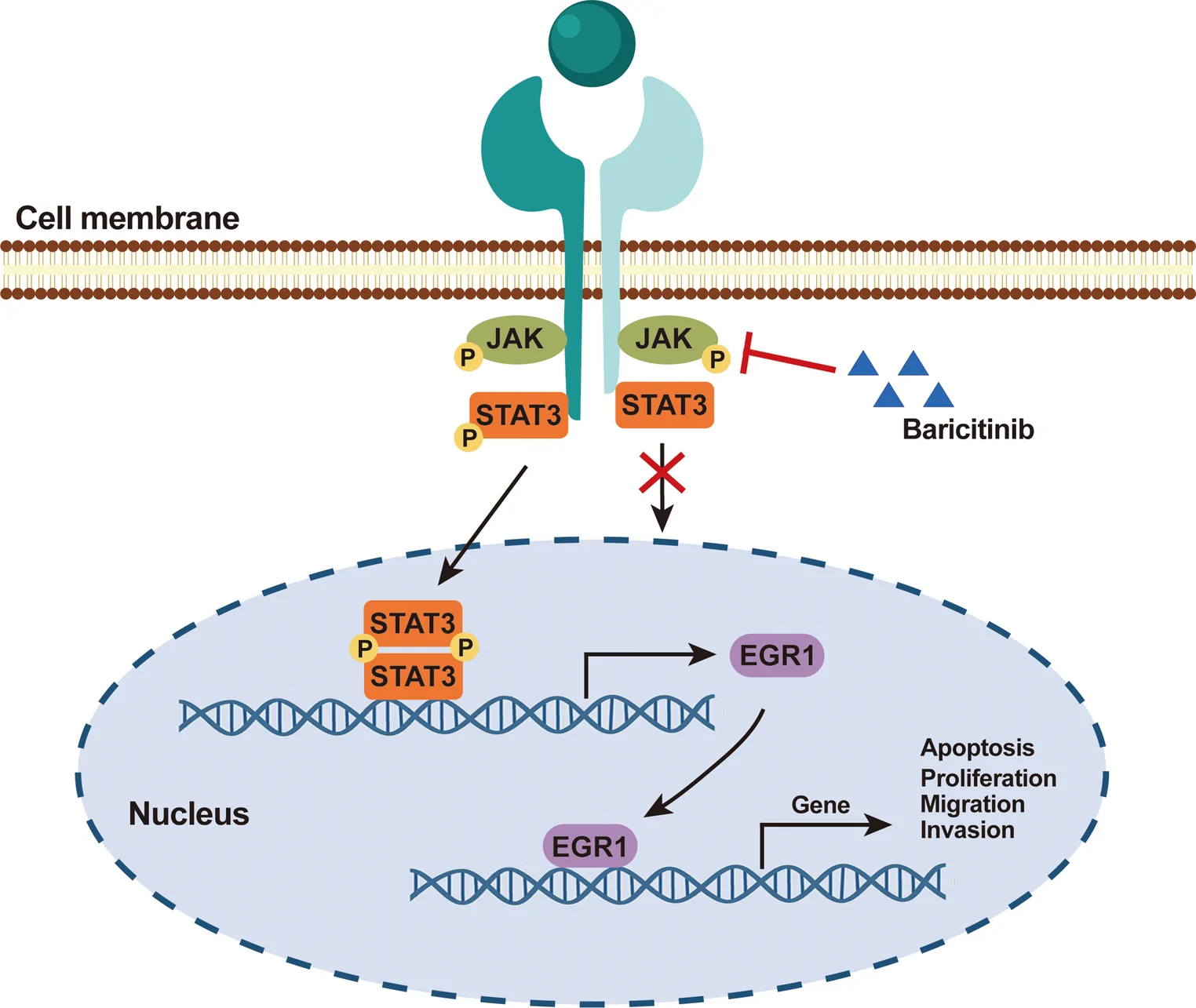
Schematic model of the proposed mechanism of action of baricitinib in RASFs.

In conclusion, our study demonstrates that baricitinib directly targets the aggressive phenotype of RASFs by inhibiting the JAK-STAT3 pathway and its key downstream effector, EGR1. This axis regulates apoptosis, invasion, and inflammatory mediator production. Our data integrate baricitinib’s immunomodulatory effects with a novel direct action on synovial pathology, proposing the JAK-STAT3-EGR1 axis as a central mechanism (Fig. 7).

## Data Availability

The datasets generated and/or analyzed during the current study are available from the corresponding author on reasonable request.
